# Exploring the Broader Benefits of Obesity Prevention Community-based Interventions From the Perspective of Multiple Stakeholders

**DOI:** 10.1007/s10728-024-00495-x

**Published:** 2024-10-03

**Authors:** J. Jacobs, M. Nichols, N. Ward, M. Sultana, S. Allender, V. Brown

**Affiliations:** 1 Global Centre for Preventive Health and Nutrition (GLOBE), Institute for Health Transformation, Deakin University, Geelong, VIC Australia; 2 Deakin Health Economics, Global Centre for Preventive Health and Nutrition (GLOBE), Institute for Health Transformation, Deakin University, Geelong, VIC Australia

**Keywords:** Cost-effectiveness, Obesity prevention, Community-based interventions, Economic analysis, Broader benefits

## Abstract

Community-based interventions (CBIs) show promise as effective and cost-effective obesity prevention initiatives. CBIs are typically complex interventions, including multiple settings, strategies and stakeholders. Cost-effectiveness evidence, however, generally only considers a narrow range of costs and benefits associated with anthropometric outcomes. While it is recognised that the complexity of CBIs may result in broader non-health societal and community benefits, the identification, measurement, and quantification of these outcomes is limited. This study aimed to understand the perspectives of stakeholders on the broader benefits of CBIs and their measurement, as well as perceptions of CBI cost-effectiveness. Purposive sampling was used to recruit participants from three stakeholder groups (lead researchers, funders, and community stakeholders of CBIs). Online semi-structured interviews were conducted, taking a constructivist approach. Coding, theme development and analysis were based on published guidance for thematic analysis. Twenty-six stakeholders participated in the interviews (12 lead researchers; 7 funders; 6 community stakeholders). Six key themes emerged; (1) Impacts of CBIs (health impacts and broader impacts); (2) Broader benefits were important to stakeholders; (3) Measurement of benefits are challenging; (4) CBIs were considered cost-effective; (5) Framing CBIs for community engagement (6) Making equitable impacts and sustaining changes—successes and challenges. Across all stakeholders, broader benefits, particularly the establishment of networks and partnerships within communities, were seen as important outcomes of CBIs. Participants viewed the CBI approach to obesity prevention as cost-effective, however, there were challenges in measuring, quantifying and valuing broader benefits. Development of tools to measure and quantify broader benefits would allow for more comprehensive evaluation of the cost-effectiveness of CBIs for obesity prevention.

**Supplementary Information:**

The online version contains supplementary material available at 10.1007/s10728-024-00495-x.

## Introduction

Obesity is a significant public health issue, which is widely regarded as an outcome of complex systems and myriad proximal and distal drivers across multiple levels of those systems [1]. Community-based interventions (CBIs) incorporate multiple strategies, across multiple settings and are developed by, and embedded in, the community [2]. This approach has been recognised as critical in creating interventions that are acceptable to stakeholders and adaptable to the local context [3]. This approach can be adapted to target specific groups in the community (e.g. children, with a focus on delivery in schools or early education settings), or be aimed at the wider population. A systematic review and meta-analysis of whole of community interventions to prevent excessive population weight gain found significant reductions in body mass index (BMI) z-score among child participants in intervention communities compared to control [4]. In addition, there is some evidence of effect of obesity prevention CBIs on other health behaviours and quality of life [5, 6]. For instance, the Whole of Systems Trial of Prevention Strategies for Childhood Obesity trial conducted in ten communities in Victoria, Australia demonstrated significantly reduced consumption of takeaway foods and improved physical, psychosocial and global health-related quality of life in intervention participants [5].

Interventions offer the best value to communities if they are both effective and cost-effective, yet establishing these metrics for complex, multi-setting CBIs for obesity prevention is challenging [2, 7, 8]. While BMI z-score is commonly the primary outcome for obesity prevention CBIs, it has been questioned whether this outcome can meaningfully measure and capture the impact of complex interventions [9]. Current economic evidence on the ‘value for money’ of CBIs for obesity prevention is usually limited to the costs and benefits related to such anthropometric outcomes (e.g. reductions in BMI), and associated direct health benefits and healthcare cost-savings [9]. Obesity prevention CBIs can have broader impacts including an increase in social connectedness, impact on climate change and community empowerment [2, 8].

Failing to include broader benefits means intervention cost-effectiveness is underestimated, yet incorporating these outcomes does not align with the widely accepted quality-adjusted life year (QALY) and disability-adjusted life years (DALY) frameworks for economic evaluation [10]. The costs and consequences of CBIs may be wide-ranging and are characterised by a lack of validated tools to measure outcomes (particularly non-health outcomes), and challenges in valuing those outcomes [10, 11].

The socio-ecological model (SEM) is a framework that has been applied and adapted across many fields, including obesity prevention [12]. This model identifies five levels; individual, interpersonal, community, environment and policy/systems, and outlines how these can interact to influence health outcomes. A recent systematic review of the outcomes and outcome measurement instruments reported in obesity prevention CBIs identified 140 unique outcomes collected or reported in CBIs, across 17 outcome domains [13]. This review identified ‘individual’ level outcomes as the most commonly reported domain, including anthropometry and body composition (e.g. BMI, waist circumference), physical activity and dietary intake [13]. Environmental measures were also regularly reported [13].Despite the significant number of outcomes reported, it was recognised that many potential impacts were rarely, or never, routinely measured, particularly those that would be considered ‘community’ or ‘policy/systems’ level of the SEM. Several reasons were discussed as to why this may be the case, including a relative lack of validated instruments, as well as lack of scope or fundings [13]. Further under-estimation of the value of CBIs may arise due to a lack of consideration of the potential impacts on family or other community members who are not directly targeted in the CBI (referred to as spill-over effects). A recent systematic review found some, albeit limited, evidence of a positive impact of CBIs on parents/caregivers of children involved in the intervention [14] although these impacts were not commonly reported.

A better understanding of the broader impacts of CBIs for obesity prevention is required, and must extend beyond those outcomes currently collected and reported [15]. This involves considering those consequences of interventions that may be harder to quantify (and thus less likely to feature in the academic literature), but which hold value in the eyes of stakeholders [7].

This qualitative study aimed to explore the views of three stakeholder groups; lead researchers, funders and community stakeholders involved in CBIs, on:

1. the health benefits and broader benefits of CBIs for obesity prevention, and how these are measured
2. the importance of broader benefits of CBIs
3. whether CBIs present a cost-effective approach for obesity prevention

## Methods

Study reporting follows the COnsolidated criteria for REporting Qualitative research (CORE-Q) guidelines for qualitative research [16] (Supplementary Table S1).

### Study Design

This was a qualitative study involving key informant interviews assessing broader benefits of CBIs for obesity prevention, with participants identified for their experience and expertise in the field. The study is informed by a constructivist epistemological stance, considering meaning is socially constructed through shared experiences and can be understood through collecting the views of participants with experience of the phenomenon under study [17].

### Research Team and Reflexivity

Three female research team members conducted the one-on-one interviews; VB (health economist), NW (health economics PhD student) and JJ (postdoctoral research fellow). Each researcher was trained in methods of qualitative research and analysis. VB has led economic evaluation of obesity prevention CBIs, JJ has experience in evaluation of large-scale obesity prevention CBIs, and NW has fieldwork experience designing and delivering CBIs.

### Participants and Recruitment

Purposive sampling was conducted, targeting three stakeholder groups: 1. Lead researchers involved in the design, conduct and/or evaluation of CBIs; 2. Funders or potential funders of CBIs, such as representatives from health departments, health promotion organisations or local government; and 3. Community leaders and members with experience or involvement in CBIs. Lead researchers involved in the design and conduct of CBIs were identified through the researchers’ knowledge and networks, and identification of investigators from peer-reviewed publications and trial registries. The interviews conducted with the funders stakeholders group were aligned with another study exploring the inclusion of climate change actions in CBIs. To reduce participant burden the interview incorporated questions for both studies in the interview schedule. Funders or potential funders of CBIs were identified by searching relevant organisations’ public websites and social media to identify appropriate stakeholders. Community leaders and members with CBI experience were identified by searching public websites and social media. Potential participants were approached through emailing publicly available email addresses outlining the study, including the plain language statement and consent form and the lead researcher’s contact details. Emails were sent by research staff who did not have working relationships with the potential participants. Participants were asked to return a consent form as confirmation of their agreement to participate. If there was no response after two weeks, one follow-up email was sent. Once a consent form was returned, the participant was contacted to arrange an interview. Interview participants were also asked to suggest other potential participants, creating a snowballing sampling method.

The proposed sample size for the semi-structured interviews followed recent guidance on reaching saturation in qualitative research, finding that saturation is typically reached in studies with relatively homogenous populations and narrowly defined objectives in 9 to 17 interviews [18]. Following this guidance we aimed to recruit 30 stakeholder participants (n = 10 from each stakeholder group). We aimed to recruit Australian and international participants to gain a range of perspectives on the benefits of CBIs to improve the external validity of results.

### Data Collection

Data were collected using semi-structured interviews as they provide participants with the opportunity to provide in-depth detail on their own individual experiences and perceptions [19]. A semi-structured interview guide was developed by two authors (VB, MS) and reviewed by all co-authors (Supplementary Table S2). The guide was designed to allow participants to discuss potential impacts of CBIs as they perceived them, and while informed by the SEM did not specifically prompt participants by SEM level to avoid leading or biased responses. The three interviewing researchers primarily conducted interviews with one stakeholder group each; CBI lead researchers (VB), funders or potential funders (NW) and community stakeholders (JJ). The researcher interviewing the CBI lead researchers had prior working relationships with some participants, however was not involved in the recruitment process and there was no coercion regarding participants’ decision to participate in the interview. The researchers conducting the funders and community stakeholders interviews had no prior relationship with the participants. All interviews were conducted online using Zoom (San Jose, CA, Zoom Video Communications Inc), and recorded and transcribed using the platform’s automated recording and transcription services. Transcripts were then checked, cleaned and de-identified by three researchers (JJ, MS, NW).

### Data Analysis

Thematic content analysis was conducted, following Braun and Clarke’s six steps for thematic analysis [19] including familiarisation, generation of initial codes, development of potential themes and sub-themes, revision and development of themes, defining and naming themes, and production of the final analysis report. Cleaned and de-identified transcripts were uploaded into QSR-NVivo 14, which was used for all coding and analysis. Codes were generated using an inductive approach by JJ and NW. In line with Braun and Clarke’s approach, it is acknowledged that two researchers are unlikely to interpret the data in the same way [20]. Therefore, once generated, codes were discussed between JJ, NW and VB and a final set of codes were agreed on by all three. All transcripts were then coded by JJ and reviewed by VB to maintain consistency and check assumptions made.

Theme development was undertaken using a deductive approach, using the SEM [12] as a guiding framework firstly by JJ, and with review and further refinement through discussion with VB. The SEM identifies five levels; individual, interpersonal, community, environment and policy/systems, and outlines how these can interact to influence health outcomes. This framework has been applied and adapted across many fields, including obesity prevention [12]. Classification of health benefits and broader benefits into groups and SEM levels was based on current literature [21] and researchers’ knowledge. Health benefits were defined as those that were directly related to an individuals’ physical or mental health. The term ‘broader benefits’ encompassed both non-health outcomes and spill-over effects. Non-health outcomes were defined as those that did not relate to a direct health outcome, but may be beneficial at a social, community or policy level [22]. Spill-over effects were defined as impacts (health or non-health) on community members (i.e. caregivers, siblings) who did not directly receive the intervention [23] (e.g. a CBI targeting children may also have a impact on their parents or caregivers). Anonymised quotes are included throughout the results to further emphasise the key themes that emerged.

## Results

A total of 112 potential participants were sent emails across 10 countries (n = 92 in Australia), between August 2022 and August 2023. Twenty-six people agreed to participate (23% response rate; 12 lead researchers; 7 funders; 6 community stakeholders). All participants engaged in one-on-one semi-structured interviews, apart from two funders who were interviewed together at their request. Interviews were conducted between August 2022 and September 2023. Participants were primarily based in Australia (n = 21), with one participant from each of New Zealand, the South Pacific, the United States, the United Kingdom and the Netherlands. The lead researchers had all been named as lead investigators on research grants that implemented and/or evaluated large obesity prevention CBIs. The funders held government funded health positions where they had some knowledge of the funding decision-making process. All community stakeholders were employed by local health or community services and had been involved in CBI design and/or implementation at the community level. Community stakeholders were also active members of their community outside of their work roles, therefore had insight in the CBIs from a community participant perspective. The length of time that participants had been involved in obesity prevention CBIs varied from 1 year to over 20 years. Interview length ranged from 28 to 56 min.

Six themes were identified across the data; (1) Participants identified health and broader benefits of CBIs; (2) Broader benefits were important to stakeholders; (3) Measurement of benefits is challenging; (4) CBIs were considered cost-effective; (5) Framing CBIs for community engagement (6) Making equitable impacts and sustaining changes—successes and challenges.

## Theme One: Participants identified health and broader benefits of CBIs

In total, participants identified six key health benefits and 15 broader benefits arising from CBIs (Table 1).

**Table 1:** Benefits identified by participants

Socio-ecological model level	Benefit identified	Example quotes	Participant group identifying benefit	Number of participants identifying benefit
*Health benefits identified*				
Individual	Changes in obesity levels	*‘reductions in BMI’* *‘reductions in obesity’*	LR, CS, F	5
Individual	Health-related behaviours	*‘eating more fruit and veggies’* *‘being more active, getting away from screens’*	LR, CS, F	8
Individual	Oral health	*‘dental health would hopefully improve’* *‘free dental health checks’*	LR, CS, F	3
Individual	Well-being or quality of life	*‘positive impacts on quality of life’* *‘measures of…mental health and well-being’*	LR, CS, F	11
Individual	Acanthosis nigricans reduction	*‘acanthosis nigricans reduction’*	LR	1
Individual	Health or food literacy	*‘elements of food literacy covered’* *‘health literacy benefits’*	LR, CS	3
*Broader benefits identified: Non-health benefits*				
Individual	Individual empowerment	*‘advocacy from community members’* *‘support people to navigate that complex system’*	LR, CS, F	5
Individual	Employment	*‘local employment in production’*	LR, F	2
Individual	Education benefits	*‘educational attainment’* *‘attendance for children at school’*	LR, CS	6
Individual/ Interpersonal	Social connections	*‘stronger connections to their community’* *‘social inclusion’* *‘change in social dynamic’*	LR, CS, F	15
Individual/ Community	Upskilling of community leaders or community members	*‘build capacity for people to use this process’* *‘adapt to other issues’* *‘peer education model’*	LR, CS, F	7
Community	Community empowerment	*‘capacity and empowerment of communities’* *‘bringing community together to solve an issue’*	LR, CS	4
Community	Community capacity	*‘organizations pivoted really quickly when COVID hit’*	LR, CS, F	6
Community	Community engagement	*‘building that connection with the community’*	LR, CS, F	10
Community/ Systems	Community networks/partnerships	*‘partnerships and levels of trust between different organizations’* *‘brought them together to work together with a common goal’*	LR, CS, F	11
Environment	Environmental/climate change	*‘reducing the number of car trips to school’* *‘creation of green space’* *‘reducing packaging waste’*	LR, CS, F	11
Environment/Systems	Food environment or food systems change	*‘growing movement of local food’* *‘changing the food systems in those communities’* *‘an all green café’*	LR, CS, F	10
Environment/ Systems	Infrastructure or physical environment changes	*‘changing the water plant’* *‘creating an environment which is conducive and encourages physical activity’* *‘built a lot of play areas for children’*	LR, CS, F	10
Policy	Economic/productivity	*‘fewer sick days’* *‘that preventive approach’* *‘more profitable than when they were selling fish and chips’* *‘families saving money on sugar sweetened beverages’*	LR, CS, F	8
Policy/Systems	Policy change or implementation	*‘policy changes the highest level’* *‘working on giving municipalities a way to ban a fast food around schools’* *‘the policy of community events that they some healthy options’*	LR, CS, F	5
*Broader benefits identified: spill-over effects*				
Interpersonal	Spill-over to other family members, community members	*‘ripple effect even on to staff’* *‘they already know some of the messages, siblings have talked about it’* *‘parents actually learn from their kids’*	LR, CS, F	12

*LR* lead researchers, *CS* community stakeholders *F* funders, *BMI* body mass index

There was variation in how the different participant groups described CBI benefits. The most common health benefits identified by all three groups were change in overweight or obesity prevalence, improved health behaviours such as physical activity, dietary intake and sedentary behaviour and mental health related. Other health benefits mentioned were oral health (all participant groups), identification of acanthosis nigricans as an early indicator of type 2 diabetes (identified by a lead researcher) and health or food literacy (identified by lead researchers and community stakeholders).

Non-health benefits arising from CBIs had wider impacts across the SEM, being classified from individual through to systems or policy level benefits. At the individual level, the three participant groups identified individual empowerment as a non-health benefit. Lead researchers and funders felt that CBIs can result in local employment opportunities and lead researchers and community stakeholders highlighted school-related outcomes, such as academic performance.

Some non-health benefits identified spanned two or more SEM levels, such as social connections (individual, interpersonal) and upskilling of community members (individual, community), which were both identified by all participants groups.

Both lead researchers and community stakeholders felt that community empowerment was an important outcome from CBIs, and all three participant groups identified that CBIs resulted in greater community capacity and community engagement. The establishment of community partnerships and networks was discussed as a key benefit of CBIs, with the ability to have an impact at both the community and systems levels of the SEM. Benefits at the environmental level included those that had a potential impact on climate change, which were identified by all participant groups. Changes to infrastructure or the physical environment as a result of a CBI, and changes to the food system were both commonly reported by all participant groups, encompassing both the environment and systems levels of the SEM. At the policy level, economic and policy benefits were identified.

The three participant groups identified that there were also spill-over effects from the CBIs, whereby benefits were experienced by family or community members not directly participating in the interventions. These spill-over effects were primarily identified as health benefits, such as children influencing parents’ or siblings’ health behaviours as a result of involvement of an intervention at school.

## Theme two: Broader benefits were important to stakeholders

All participants were able to identify at least two broader benefits that had arisen from CBIs, expressing that these were very important outcomes of CBIs.

Participants from the three stakeholder groups identified the establishment of community networks and relationships through the CBI process as being one of the most important and highly regarded outcomes from CBIs.

Strengthening those partnerships and connections. Because that's, well that's the foundation of all of our work really. You can have the best program ever, but if you don't have a connection with the setting or the community, it's of no use. Community Stakeholder #4

Participants further elaborated that in many communities these networks and partnerships had moved forward to address other issues in the community, particularly throughout the COVID-19 pandemic.

Through the process of the establishment of the CBIs, definitely brought a number of community groups together that that wouldn't generally have the opportunity to network or connect and we've seen in a number of spaces that those relationships have been maintained and, you know, progressed onto other actions and pieces of work in other realms that wouldn't have otherwise happened. Community Stakeholder #5

At a more individual level, stakeholders felt that CBIs had resulted in greater social inclusion and social connectedness, and that community members were participating in activities more regularly. Despite being considered important, participants reported that these broader outcomes were often not measured, and were their perceptions of the benefits, and anecdotally reported from community members or participants.

## Theme Three: Measurement of benefits is challenging

Participants highlighted the many challenges with selecting, collecting and interpreting measures of both health and broader benefit outcomes used to evaluate the impact of complex interventions.

### Challenges in Measuring Health Benefits

Many participants discussed the use of BMI z-score as a primary outcome and whether this measure can adequately capture and represent the impact of complex interventions. However, as several participants pointed out, BMI z-score allows for comparison between studies, and is able to be reliably and relatively easily measured in CBI participants.

I spent a long time thinking about BMI z-score. I don't think it's a fantastic primary outcome, but this level we have got 1,000 children, and …… if we want the findings to be comparable to other research, kind of, we're kind of stuck with it. Lead Researcher #8

Participants also discussed the challenges of measurement tools to be able to detect changes that can be attributed to the intervention being evaluated. This difficulty can be due to the measured impacts being distal to the intervention action.

Things like community gardens. You know, it's actually fairly difficult to show any changes in healthy eating as a result of that kind of intervention. Funder #2

Due to the nature of CBIs, which involve multiple settings and approaches, it is also challenging to untangle the impacts of interventions, and attribute success when there are many contributing factors involved.

How do you attribute an outcome to one specific intervention…it's a bit difficult to do that, particularly when you've got multiple players’ Lead Researcher #11

Further discussion of the challenges in attribution of success, related to whether changes were due to the interventions, or changes in attitudes and behaviours over time at a societal level. One lead researcher discussed how water consumption is now a much more normal behaviour than in previous decades, therefore it is difficult to know how much of this behaviour change has been due to their targeted program, or changes in social norms.

I think it's hard to, for stakeholders to exactly pinpoint what other things contributed, so they can tell, well, we did that… I think it's often got, also got to do with, you know, just general developments in society. Lead Researcher #10

Participant burden was another key consideration in determining how health benefits from CBIs were measured. In particular, lead researchers discussed the need to balance collecting enough information to be able to effectively evaluate an intervention with not overburdening CBI participants.

We've kept our outcomes to an absolute minimum, again, because……. we didn't want to risk losing people to the primary outcome. So we’ve actually prioritized that. Lead Researcher #8

### Challenges in Measuring Broader Benefits

It was widely acknowledged that measurement of broader benefits was a challenge, and was rarely undertaken, for a wide range of reasons. A common sentiment was a lack of existing tools to measure many of these outcomes, in particular those at the environment, systems and policy level, which were often incidentally observed.

It would be good to have something where we could measure what we actually do as a collaborative…… it doesn't exist. Community Stakeholder #4

Further to this, limited funding or capacity made it difficult to either develop appropriate tools or modify existing tools to the community environment. Participants did highlight that there were some tools or approaches used to measure some community level outcomes, such as social network analysis, community readiness to change scales and tracking of intervention actions. However, these were often reported to be time-consuming, placed significant burden on the participants and were impacted by staff turnover.

We looked at community readiness to change as a tool…. it was very burdensome … and too technical to score. Lead Researcher #12

Social network analysis, we collected that as well…..but the problem was, is that so much staff turn over that you couldn't actually follow up a lot of the people, and with more and more restrictions around privacy rules, and how you contact people……became a real challenge to follow up people or to find out who to follow up. Lead Researcher #12

The needs of funding bodies were also a key consideration in decisions on measurement of broader benefits. Participants identified that funding decisions and measures of success still primarily relate to intervention impacts on core health outcomes such BMI z-score, physical activity levels and dietary intake. This hinders the ability of those designing and delivering CBIs to expand their outcome measures within the limited timeframes and capacity.

We just don't have the capacity or time to do that, and it's not part of… the remit ....no one's going to fund us to measure it, like it's just too far outside the things people will fund. Lead Researcher #4

As was the case for measurement of health outcomes, participant burden was a key consideration and challenge to measurement of broader benefits. This was often discussed in relation to the community members participating in the design and implementation stages of the intervention;

Uptake through networks is promising. But at the moment… it's very cumbersome. It's very time consuming and big burden on the participants to record all their connections, and how much they've interacted with various people and about what. Lead Researcher #3

‘We're trying to have the short and sharp surveys to not burden participants, and it's really like how many serves of fruit and veg did you eat… I don't feel like we're really looking at knowledge, attitudes, behaviours, you know, intentions, awareness about things, confidence in cooking’ Lead Researcher #12

## Theme Four: CBIs were considered cost-effective

Overall, participants felt that CBIs were a cost-effective approach to obesity prevention, agreeing that the benefits of the intervention outweigh the costs of running the CBIs. Several discussed the cost-effectiveness in comparison to the alternative of not preventing chronic disease.

Do the benefits outweigh the costs? Definitely… one of the things that I think you need to factor into an analysis like that, is the cost of doing nothing which health systems are wearing all of the time….. due to a lack of primary prevention community-based initiatives. Lead Researcher #9

Participants also expressed that CBIs were cost-effective because a significant portion of the resources used was a result of community members volunteering their time and resources to design and implement interventions that were most appropriate for their community.

The costs are actually minimal….you know, some health promotion staff in local government…. in health services to come together as partners and be that kind of background facilitator is pretty damn low compared to the action and the momentum within the communities. Community Stakeholder #1

‘They're cost-effective because you're drawing on as much as you can. You're drawing on existing resources and communities’ Lead Researcher #9

While identifying that the costs were low, some participants felt that potentially more funding could be directed towards CBI implementation, as reliance on existing resources can lead to poor sustainability, and the intervention being overshadowed by competing community interests.

… initially, we were sort of …… selling this processes as it will be embedded and become usual practice, where I actually still feel you actually need dedicated and protected resources. Lead Researcher #12

Participants felt CBIs were cost-effective, though commonly reported being unaware of evaluations demonstrating CBI cost-effectiveness.

I think that is really hard to, a hard thing to, to figure out, and also because of these broader benefits. So what is the benefit from collaborating with other policy sectors?’ Lead Researcher #10

‘What we're doing is trying to reorient existing funding, and to operate in a different way. You know how you measure that and how that's had some effect, that rather falls outside the cost-effectiveness methodology’ Lead Researcher #3

## Theme Five: Framing CBIs for community engagement

A theme discussed by all participant groups was how CBIs are framed to maximise community engagement and minimise adverse outcomes. In particular, there was a more recent trend away from using the term ‘obesity prevention’ due to negative connotations and perceived stigma associated with such language and with weight-focused outcomes. Participants observed a move towards language and intervention focus that was more about holistic health promotion, or the healthy eating, physical activity and quality of life aspects of interventions.

I think it's more, it's more palatable in the community. As soon as you bring in that obesity, it just turns people off. Community Stakeholder #3

….thinking about most programs in that setting [schools] there is often promotion of the co-benefits rather than a weight focus. Funder #1

There was discussion that while this shift is happening at a societal level, it may conflict with the key outcome measurements that are currently identified as being needed to show effectiveness, such as BMI z-score (requiring measurement of participant heights and weights), to obtain funding for future similar programs. This tension between acceptability to the community, while still appeasing the funding bodies was expressed by a number of participants.

Most of the grants…. have been focused on obesity or the weight status of children as the primary outcome. Lead Researcher #9

‘I hope that we can… continue to promote this approach, particularly to the [government] department ….their narrative tells us that this is a great approach, but their funding restrictions say otherwise. So you know, they're very descriptive with the kind of programs and things like that that they want us to be delivering, which flies in the face of community needs in some instances’ Community Stakeholder #4

## Theme six: Making equitable impacts and sustaining changes—successes and challenges

There were differing views on whether CBIs achieve equitable outcomes within communities. Some participants felt that due to the broad nature of the CBIs, with focus on changing the environments, that there was a greater chance of a more equitable impact from the interventions.

Because a lot of the work is more around environments and choices…. you hope that there's a lot more universal stuff to that as opposed to delivering a program to a certain amount of people. So, yeah, I suppose that was kind of the comforting factor to that that if you know changing the whole community system, it's a benefit for all. Community Stakeholder #1

Some participants expressed, however, that the benefits of CBIs were not always equally distributed across community groups, particularly across the socio-economic spectrum. While focusing on schools and early learning centres helped to deliver the messages and programs universally, there was concern from participants that other elements of the interventions meant that impacts were not equitably distributed.

I think schools are great, because we obviously get access to the vast majority of families and kids. But with the water stuff primarily being focused in sports clubs, yeah, I'm aware that there's some pretty huge barriers there, it's not cheap to play sport. So I think that there probably is a discrepancy there in regards to socio-economics. Community Stakeholder #5

The potential differential impact was generally hypothesised by participants, however they were not generally aware of whether benefits had been formally measured across different groups within the communities, or how this could be measured.

In addition to having equitable impacts, a number of participants discussed the difficulty in sustaining positive changes made in communities, particularly in regards to the primary health outcomes, beyond the initial intervention period.

Multiple reports of short-term reductions in BMI, so that's really exciting and really promising. But we don't sustain, we don't see that the efforts and the outcomes are sustained. Lead Researcher #1

Much of this difficulty in sustaining changes was tied to funding cycles, where efforts were not able to continue once the initial funding period finished.

That's probably the single, most negative feature associated with some of this work……That lack of a continuity of funding and certainty of funding and security of funding, it has a major impact on successes and outcomes of these programs. Lead Researcher #11

However, it was the broader benefits, such as the development of partnerships and networks within communities, that were often seen as the key components that were required if changes were going to be sustained within the communities.

Institution building is important, not only to the success of the program, but perhaps more importantly to the sustainability’ Lead Researcher #2

## Discussion

This study explored perceptions of three stakeholder groups on the health and broader benefits of obesity prevention CBIs, and whether these were being measured or tracked in meaningful ways. This study also sought to determine the relative importance of the broader benefits to these stakeholders, and whether different participant groups perceived CBIs to be cost-effective. Participants identified many health and broader benefits arising from CBIs, however quantifying broader benefits and sustainability of outcomes were identified as challenges despite participants reporting they felt CBIs were a cost-effective approach to obesity prevention. Perspectives from the three participant groups highlighted that there are a number of similar issues and challenges present for stakeholders involved at different points in the CBI process, but these may be perceived as impacting in varying ways depending on a stakeholder’s role and experience.

The common individual health benefits identified by our participants were broadly in line with those found in an extensive systematic review of outcomes and outcome measures used in obesity prevention CBIs [13]. However, in contrast to the systematic review findings, many of our participants also identified benefits that would be considered to be at the community or policy/systems levels of the SEM, such as creation of community networks, community empowerment, influence on local policies and changing local food systems.

Participants had various views on the use of anthropometry (most commonly BMI and BMI z-score) as a measure of success of interventions. Measuring BMI is generally the most common used metric of success in obesity prevention CBIs, and therefore the most comparable across studies, with recent reviews finding 78% [24] and 91% [13] of complex childhood obesity prevention interventions reported BMI. It is to be expected that BMI is widely used as a primary outcome in obesity-focused interventions, however participants noted there were problems in measuring and reporting BMI-related outcomes, in the context of a move away from framing and communicating interventions explicitly around weight, in preference for promoting health more generally. There remains, however, a pragmatic need to continue to monitor BMI at a population level to track changes, with this measure also being reasonably low-cost, accessible and allowing for comparison with other studies [25]. Additionally, while participants saw significant value in the broader benefits generated by CBIs, funding bodies often still require traditional evidence of obesity prevention, usually measured by BMI, to show the success of programs and be competitive for future funding.

Despite the perceived value of broader benefits to stakeholders, this study found they were rarely measured, a finding re-enforced by a study of systematic reviews, that showed non-health outcomes were only incorporated into 8% of 494 evaluations [22], often due to a lack of appropriate measurement tools [22]. The results of our interviews were broadly in line with the documented methodological challenges of economic evaluations of public health interventions proposed by Weatherly et al. [10], who identified measurement and value of non-health outcomes as one of four key challenges. These issues are not unique to obesity prevention, also being reported in alcohol [26] and diabetes prevention [27]. Without being able to measure these important broader benefits, the value of CBIs are likely currently being under-estimated, a sentiment supported by a recent systematic review including 13 interventions specifically focused on obesity prevention CBIs in children [9]. Further potential under-estimation of the benefits of obesity prevention CBIs may occur due to the low number of studies reporting spill-over effects in family or other community members who were not directly targeted by the intervention [14]. Challenges in developing new tools to capture broader benefits in public health interventions were discussed by a number of our participants. Similarly, van Mastrigt et al [11] explored expert views on non-health benefits of health promotion interventions, finding a majority of interviewees supported the development of an instrument that captured these benefits in economic evaluations, however some participants queried the usefulness and feasibility of such a tool, and whether it would be able to capture meaningful information.

Previous studies of non-health outcomes have had a greater focus on individual factors [28, 29] however, participants in this study identified benefits across the five levels of the SEM with the creation of partnerships and networks in communities being a commonly discussed broader benefit. Best-practice guidelines for the conduct and reporting of cost-effectiveness analyses in health and medicine recommend evaluations from both a health care and a societal perspective (i.e. accounting for all costs and effects, regardless of who they are borne by) [15]. The challenges in measurement, quantification and valuation of broader benefits identified in this study may help explain why these broader costs and benefits are not generally incorporated into cost-effectiveness analyses [9]. The complexities of undertaking economic evaluations of CBIs for obesity prevention are well-recognised, however methods such as return-on-investment (ROI), cost–benefit analysis (CBA) and/or cost-consequence analysis (CCA) have recently been recommended in order to better incorporate broader or inter-sectoral costs and benefits into analyses [9, 22]. It is clear from our study findings however that the development of methods for measurement and valuation of particularly non-health outcomes will play an important role in moving the field forward, especially when health gains may not be the only benefit of an intervention.

Our study shows that the broader benefits were key in creating sustainable changes in communities, which aligns with the framework for public health program sustainability [30] whereby both ‘partnerships’ and ‘organisational capacity’ were key domains in achieving sustainable outcomes. Our study supported the further domains of this framework [30], finding ‘program evaluation’ and ‘funding stability’ were essential for sustainability. Shelton et al. (2023) argues that sustainability and equity are closely aligned, with populations that face barriers to optimal health also experiencing enhanced sustainability challenges [31]. We found little evidence that participants were aware of CBIs being evaluated according to socio-economic metrics. This is consistent with the most recent Cochrane review on obesity prevention in children, which found only 11 of the 153 studies reported outcomes according to socioeconomic position [32].

This study aimed to better understand the range of potential benefits of CBIs from the perspective of different stakeholders, and reported the outcomes most commonly identified by stakeholder group. Given the broad range of potential outcomes identified, future work should explore stakeholder priorities or preferences for the most important outcomes from CBIs, and recommend instruments for measuring them. This research could be undertaken as part of a core outcome set (COS) development process using methods such as Delphi ranking [33] (as have been done in other areas of obesity prevention, for example [34, 35],); or quantitatively estimating preferences for outcomes using methods such as discrete choice experiment (as have been undertaken in other areas of health, for example [36]).

One caution in interpreting these results is the over-representation of researchers compared to funders and community stakeholder participants in the interviews, which may have been due to this group’s interest and knowledge in this particular area. However, the results, which showed strong alignment of views across stakeholder groups increases confidence that the resulting bias may be minimal. Despite invitations to an international cohort, a majority of participants were based in Australia, therefore the results may reflect the Australian situation more than other countries. Australia has been a leader in the development and implementation of CBIs [4], which may have also contributed to the high proportion of Australian participants. There was consistency, however, between our results and the existing international literature, which highlights common issues [22]. Further, these sampling discrepancies may have been exacerbated by our snowballing recruitment method, which can result in reduced diversity of participants, a common issue with this approach [37]. We also did not compensate participants for their time which may have impacted recruitment.

While the community stakeholders interviewed were also active members within the communities, a further limitation of this study was not including interviews with other beneficiaries of the interventions (e.g. families/children in the communities). The broad, whole-of-community nature of the CBI approach means that individual are not ‘recruited’ to participate as such, and many community members may not consider themselves ‘participants’. For example, two large CBIs that have been conducted in Victoria, Australia [5, 38] involved changes across many levels in the community, however the outcomes were only measured in school children. Additionally, our recruitment method (using publicly available information) also limited our ability to recruit community participants. However, this is an important future area of research to further investigate the views of those who have been participants in CBIs, which will require a different recruitment approach. There was some cross-over in the CBIs that some of the lead researchers and community stakeholders have been involved in, which therefore may have reduced the diversity of experiences, and resulted in similar discussions and issues raised. Further to this, we did not collect nuanced information on the types or contexts of the CBIs that participants had been involved in, so detailed analysis according to these categories was not possible.

## Conclusions

This study has highlighted a significant range of benefits arising from obesity prevention CBIs, and particularly the importance of the broader benefits arising from CBIs, from the perspective of a range of stakeholder groups. The current lack of tools to measure these broader benefits, and incorporate them in economic evaluations, is likely resulting in underestimation of the impacts and cost-effectiveness of CBIs. The range of broader benefits identified by stakeholders points to important areas of future research to develop low burden, but meaningful, measurement methods and tools, and incorporate these results into our understanding of the full impacts of obesity prevention CBIs.

## Supplementary Information

Below is the link to the electronic supplementary material.

Supplementary file1 (DOCX 23 KB)
