# Supplementary material for: Exploring the Broader Benefits of Obesity Prevention Community-based Interventions From the Perspective of Multiple Stakeholders
Source: Health Care Anal. 2024 Oct 3;33(2):151–72. doi: 10.1007/s10728-024-00495-x (PMC12052814; doi:10.1007/s10728-024-00495-x)
Supplement: Supplementary file 1 — Supplementary file1 (DOCX 23 KB) [file 10728_2024_495_MOESM1_ESM.docx]

Journal: Health Care Analysis

*Title: Exploring the broader benefits of obesity prevention community-based interventions from the perspective of multiple stakeholders.*

Authors: Jacobs J^1^, Nichols M^1^, Ward N^2^, Sultana M^2^, Allender, S^1^, Brown V^2^

1. Deakin University, Geelong, Australia, Global Centre for Preventive Health and Nutrition (GLOBE), Institute for Health Transformation.

2. Deakin University, Geelong, Australia, Deakin Health Economics, Global Centre for Preventive Health and Nutrition (GLOBE), Institute for Health Transformation.

Corresponding author:

Jane Jacobs

email: [jane.jacobs@deakin.edu.au](mailto:jane.jacobs@deakin.edu.au)

ORCID: 0000-0002-3722-9672

**Supplementary Table S1 - CORE-Q checklist (Tong et al. 2007)**

| **No.** | **Item** | **Guide questions/description** | **Page no. in manuscript** |
| --- | --- | --- | --- |
| Domain 1: Research team and reflexivity | | | |
| 1 | Interviewer/facilitator | Which author/s conducted the interview or focus group? | Pg. 6 |
| 2 | Credentials | What were the researcher's credentials? E.g. PhD, MD | Pg. 6 |
| 3 | Occupation | What was their occupation at the time of the study? | Pg. 6 |
| 4 | Gender | Was the researcher male or female? | Pg. 6 |
| 5 | Experience and training | What experience or training did the researcher have? | Pg. 6 |
| Relationship with participants | | | |
| 6 | \|  \| Relationship established \| \| --- \| --- \| | Was a relationship established prior to study commencement? | Pg. 7 |
| 7 | Participant knowledge of the interviewer | What did the participants know about the researcher? e.g. personal goals, reasons for doing the research | Pg. 7 |
| 8 | Interviewer characteristics | What characteristics were reported about the interviewer/facilitator? e.g. Bias, assumptions, reasons and interests in the research topic | Pg. 7 |
| Domain 2: study design | | | |
| Theoretical framework | | | |
| 9 | \|  \| Methodological orientation and Theory \| \| --- \| --- \| | What methodological orientation was stated to underpin the study? e.g. grounded theory, discourse analysis, ethnography, phenomenology, content analysis | Pg. 6 |
| Participant selection | | | |
| 10 | Sampling | How were participants selected? e.g. purposive, convenience, consecutive, snowball | Pg. 6-7 |
| 11 | Method of approach | How were participants approached? e.g. face-to-face, telephone, mail, email | Pg. 7 |
| 12 | Sample size | \|  \| How many participants were in the study? \| \| --- \| --- \| | Pg. 9 |
| 13 | \|  \| Non-participation \| \| --- \| --- \| | How many people refused to participate or dropped out? Reasons? | Pg. 9 |
| Setting | | | |
| 14 | \|  \| Setting of data collection \| \| --- \| --- \| | Where was the data collected? e.g. home, clinic, workplace | Pg. 8 |
| 15 | Presence of non-participants | Was anyone else present besides the participants and researchers? | Pg. 9 |
| 16 | Description of sample | \|  \| What are the important characteristics of the sample? *e.g. demographic data, date* \| \| --- \| --- \| | Pg. 9 |
| Data collection | | | |
| 17 | Interview guide | Were questions, prompts, guides provided by the authors? Was it pilot tested? | Pg. 7 and Supplementary File 2 |
| 18 | Repeat interviews | Were repeat interviews carried out? If yes, how many? | n/a |
| 19 | Audio/visual recording | Did the research use audio or visual recording to collect the data? | Pg. 8 |
| 20 | Field notes | Were field notes made during and/or after the interview or focus group? | n/a |
| 21 | Duration | What was the duration of the interviews or focus group? | Pg. 9 |
| 22 | Data saturation | \|  \| Was data saturation discussed? \| \| --- \| --- \| | Pg. 7 |
| 23 | \|  \| Transcripts returned \| \| --- \| --- \| | Were transcripts returned to participants for comment and/or correction? | n/a |
| Domain 3: analysis and findings | | | |
| Data analysis | | | |
| 24 | Number of data coders | How many data coders coded the data? | Pg. 8 |
| 25 | Description of the coding tree | Did authors provide a description of the coding tree? | n/a |
| 26 | Derivation of themes | Were themes identified in advance or derived from the data? | Pg. 8 |
| 27 | Software | What software, if applicable, was used to manage the data? | Pg. 8 |
| 28 | Participant checking | Did participants provide feedback on the findings? | n/a |
| Reporting | | | |
| 29 | Quotations presented | Were participant quotations presented to illustrate the themes / findings? Was each quotation identified? e.g. participant number | Pg. 10-19 & Table 1 |
| 30 | Data and findings consistent | Was there consistency between the data presented and the findings? | Results and discussion (pg. 10 – 23) |
| 31 | Clarity of major themes | Were major themes clearly presented in the findings? | Pg 10 – 17 (Themes 1 -4) |
| 32 | \|  \| Clarity of minor themes \| \| --- \| --- \| | Is there a description of diverse cases or discussion of minor themes? | Pg. 17 -19 (Theme 5) |

**Supplementary File S2 – Semi-structured interview guide**

Thanks for taking the time to talk with me today.

This interview will take no longer than 60 minutes.

As background, the aim of the PRECIS project is to identify and quantify the broader impacts (or co-benefits) of community-based obesity prevention interventions, so that we can estimate the impact of these additional benefits on overall cost-effectiveness. We are conducting a series of interviews with different stakeholder groups, so that we can understand some of the potential broader benefits of community-based obesity prevention interventions – from your own perspective.

You were identified as a key stakeholder to community-based obesity prevention interventions, due to your involvement with …… [add here, relevance to interview participant].

The purpose of the interview today is to hear your experience, knowledge and thoughts on the range of potential benefits of community-based childhood obesity prevention interventions. Are you happy for us to record the interview to ensure we have an accurate record of your comments and for the purposes of analysis? We will not identify any individuals in reporting of the interview findings.

Do you have any questions before we begin?

As background, community-based-interventions (CBIs) emphasise community-focused interventions, following socio-ecological approaches that employ multiple interventions, and include a range of different people and organisations to promote health and wellbeing of the population.

Today we would like you to think about all of the direct and indirect benefits that potentially arise from the community-based obesity prevention intervention(s) you have experience with.

These could be a range of benefits – perhaps they are health benefits, or they could be economic benefits, or community benefits and so on.

By benefits we mean anything of value that you see as a result of community-based obesity prevention interventions happening in your community– whether you are talking about monetary value, economic value or perhaps other things of value (e.g. social or environmental value).

Do you have any questions?

1. Would you please tell us about your experience with childhood obesity CBIs?
2. Perhaps if we talk about the key health benefits that you see are a result of community-based childhood obesity prevention interventions first? From your experience, what would you say are the health benefits of CBIs, if any?
3. Do you think these health benefits were experienced by all in your community, or do you think benefits might differ between different groups of individuals?
4. Now I would like you to think more broadly, about whether you think there are any other benefits from CBIs? So benefits that aren’t necessarily to do with health.

Can you tell me what some of those benefits might be, if any?

1. Thinking about all of the potential benefits we have discussed today, what do you think is the most important co-benefit arising from childhood community-based obesity preventions outside the primary or secondary study objectives, from the perspective of:

- Individual children
- Households and families
- Communities as a whole
- Organisation stakeholders probe only: Organisations involved in health promotion and intervention

Why do you think that?

1. Do you think the benefits of CBIs outweigh the costs of running the intervention? Yes/no – why?
